# A 3D-CNN model with CT-based parametric response mapping for classifying COPD subjects

**DOI:** 10.1038/s41598-020-79336-5

**Published:** 2021-01-08

**Authors:** Thao Thi Ho, Taewoo Kim, Woo Jin Kim, Chang Hyun Lee, Kum Ju Chae, So Hyeon Bak, Sung Ok Kwon, Gong Yong Jin, Eun-Kee Park, Sanghun Choi

**Affiliations:** 1 School of Mechanical Engineering, Kyungpook National University, 80 Daehak-ro, Buk-gu, Daegu, 41566 Republic of Korea; 2 Department of Internal Medicine and Environmental Health Center, School of Medicine, Kangwon National University Hospital, Kangwon National University, Chuncheon, Republic of Korea; 3 Department of Radiology, College of Medicine, Seoul National University, Seoul National University Hospital, Seoul, Republic of Korea; 4 Department of Radiology, College of Medicine, The University of Iowa, Iowa City, Iowa USA; 5 Department of Radiology, Research Institute of Clinical Medicine of Jeonbuk National University–Biomedical Research Institute of Jeonbuk National University Hospital, Jeonju, Republic of Korea; 6 Department of Radiology, Kangwon National University Hospital, Kangwon National University School of Medicine, Chuncheon, Republic of Korea; 7 Department of Medical Humanities and Social Medicine, College of Medicine, Kosin University, Busan, Republic of Korea

**Keywords:** Imaging, Computational models, Image processing, Biomedical engineering

## Abstract

Chronic obstructive pulmonary disease (COPD) is a respiratory disorder involving abnormalities of lung parenchymal morphology with different severities. COPD is assessed by pulmonary-function tests and computed tomography-based approaches. We introduce a new classification method for COPD grouping based on deep learning and a parametric-response mapping (PRM) method. We extracted parenchymal functional variables of functional small airway disease percentage (fSAD%) and emphysema percentage (Emph%) with an image registration technique, being provided as input parameters of 3D convolutional neural network (CNN). The integrated 3D-CNN and PRM (3D-cPRM) achieved a classification accuracy of 89.3% and a sensitivity of 88.3% in five-fold cross-validation. The prediction accuracy of the proposed 3D-cPRM exceeded those of the 2D model and traditional 3D CNNs with the same neural network, and was comparable to that of 2D pretrained PRM models. We then applied a gradient-weighted class activation mapping (Grad-CAM) that highlights the key features in the CNN learning process. Most of the class-discriminative regions appeared in the upper and middle lobes of the lung, consistent with the regions of elevated fSAD% and Emph% in COPD subjects. The 3D-cPRM successfully represented the parenchymal abnormalities in COPD and matched the CT-based diagnosis of COPD.

## Introduction

Instances of chronic obstructive pulmonary disease (COPD), a respiratory disease related to pulmonary airflow obstruction, are rising globally^1^. COPD causes several dangerous lung phenotypes, such as emphysema, chronic bronchitis, and even lung cancer. According to the 2020 chronic obstructive lung disease report, COPD has been the fourth leading cause of death, killing more than three million people (6% of the deaths worldwide) in 2012^2^. Quantitative analyses based on computed tomographic (CT) images have explored the airway trees, lung parenchyma abnormalities, and other features related to COPD^3–5^. These CT findings are useful for clarifying the lung anatomical features, enabling the prevention, early diagnosis, and management of COPD.

In regards to CT-based imaging findings, COPD is characterized by emphysematous lung, airway narrowing, functional small-airway disease, and reduced lung deformation^5^. In clinics, forced expiratory volume in 1 s (FEV1) and percentage of predicted value along with FEV1/forced vital capacity (FVC) have been used as criteria to identify the severity of COPD^6^. Such pulmonary function tests (PFTs) are convenient and inexpensive, but are not always recommended for treatment decisions due to poor stratification^2,7^. COPD risk can be more accurately stratified from visual CT data^5^. Specific features extracted from visual CT scans, such as lung parenchyma, airways, and pulmonary vessels, are effective screens for COPD. However, the visual assessment of COPD from large CT volumes is subjective, and accurately detecting COPD across large populations without manual extraction or without reviewing the specific clinical or radiographic features is burdensome to physicians^8,9^.

An accurate computer-aided detection system is essential for an efficient and cost effective COPD screening workflow. Recently, deep-learning-based methods have gained popularity in medicine, owing to their power and flexible use of the available data^5^. A deep convolutional neural network (CNN) is a deep-learning approach that automatically extracts features from data. Advances in CNNs have greatly improved the performance of image classification and detection^10–13^. A CNN can learn representative features from the data with multiple levels of abstraction and thus the design, extraction, and selection of handcrafted features are unnecessary^14^. CNN techniques can potentially improve COPD risk modeling in the absence of pulmonary function tests (PFTs) or visual assessment of lung parenchyma. Some existing machine learning algorithms can recognize or distinguish COPD in CT images, but have not been studied in detail. Unfortunately, the image feature layers trained for classification by a deep neural network remain unknown^15^. This problem might be resolved by gradient-weighted class activation mapping (Grad-CAM), which produces visual explanations from a CNN, allowing visualization of the areas focused by the CNN^15,16^.

Recently, the classification, detection, and segmentation performances of CNNs in COPD detection have significantly improved. Using CNN models and four canonical CT slices at predefined anatomic landmarks in the COPDGene and ECLIPSE testing cohorts, González et al.^17^ achieved a COPD detection accuracy of 0.773. Du et al.^18^ applied a CNN on 2D multi-view snapshots of a 3D lung-airway tree, and classified COPD and non-COPD cases. They reported an accuracy of 88.6% on grayscale snapshots. Feragen et al.^19^ applied a support vector machine (SVM) to the airway tree information of 1996 subjects, including 893 with COPD. Their highest accuracy was 64.9% in COPD classification tasks. Bodduluri et al.^20^ evaluated the ability of supervised learning to distinguish COPD patients from non-COPD subjects, applying image registration metrics to biomechanical, density, and texture feature sets. The best area under the curve (AUC) was 0.89 on texture feature sets.

In COPD subjects, airway structure and parenchymal function have been assessed with structural and functional variables obtained from quantitative computed tomography (QCT) of lung, such as luminal diameter, wall thickness, air trapping (or functional small-airway disease), and emphysema^21^. Air trapping in COPD at expiration can characterize small-airway narrowing/closure at the parenchymal level, but may contain some portion of emphysema at inspiration^6^. Accordingly, Galban et al.^4^ introduced parametric-response mapping (PRM), which dissociates the air-trapping effects of mixed air trapping and emphysema. Using this approach, we can characterize three features at the voxel level: emphysema (Emph), functional small-airway disease (fSAD), and normal lungs. Using expiration and inspiration CT scans and a voxel-wise image analysis technique, we can accurately distinguish COPD imaging phenotypes by visually assessing the PRMs of fSAD and Emph^6^.

We hypothesize that a 3D-CNN with a PRM input (3D-cPRM) can represent the abnormalities of lung parenchyma and predict clinically relevant outcomes in COPD subjects without pre-specifying the features of interest. To test this hypothesis, we investigate whether the newly trained PRM imaging features can identify COPD. We also evaluate the potential correlations between PRM and 3D-CNN in patients with COPD. Our proposed approach was implemented as follows. First, we visualized the 3D PRM model combining fSAD, Emph, and normal portions in the lung by a mass-preserving image registration method. This visualization step is of major clinical significance, as clinicians can easily observe disease alterations in the lung parenchyma captured in one frame. Second, we input the CT-based imaging variables to a 3D fully convolutional architecture, thus realizing volume-to-volume learning and inference. The specially designed 3D-CNN model with PRM was hyper-parametrized for filter-size, batch size, learning rate, and others, and further optimized for COPD identification by the Adam algorithm. Applying Grad-CAM, we finally highlighted the important regions in the images for predicting healthy control (non-COPD) or COPD. To our knowledge, our work is the first to use deep 3D-CNN and CT-based PRM to COPD classification.

## Results

**Figure 1:**
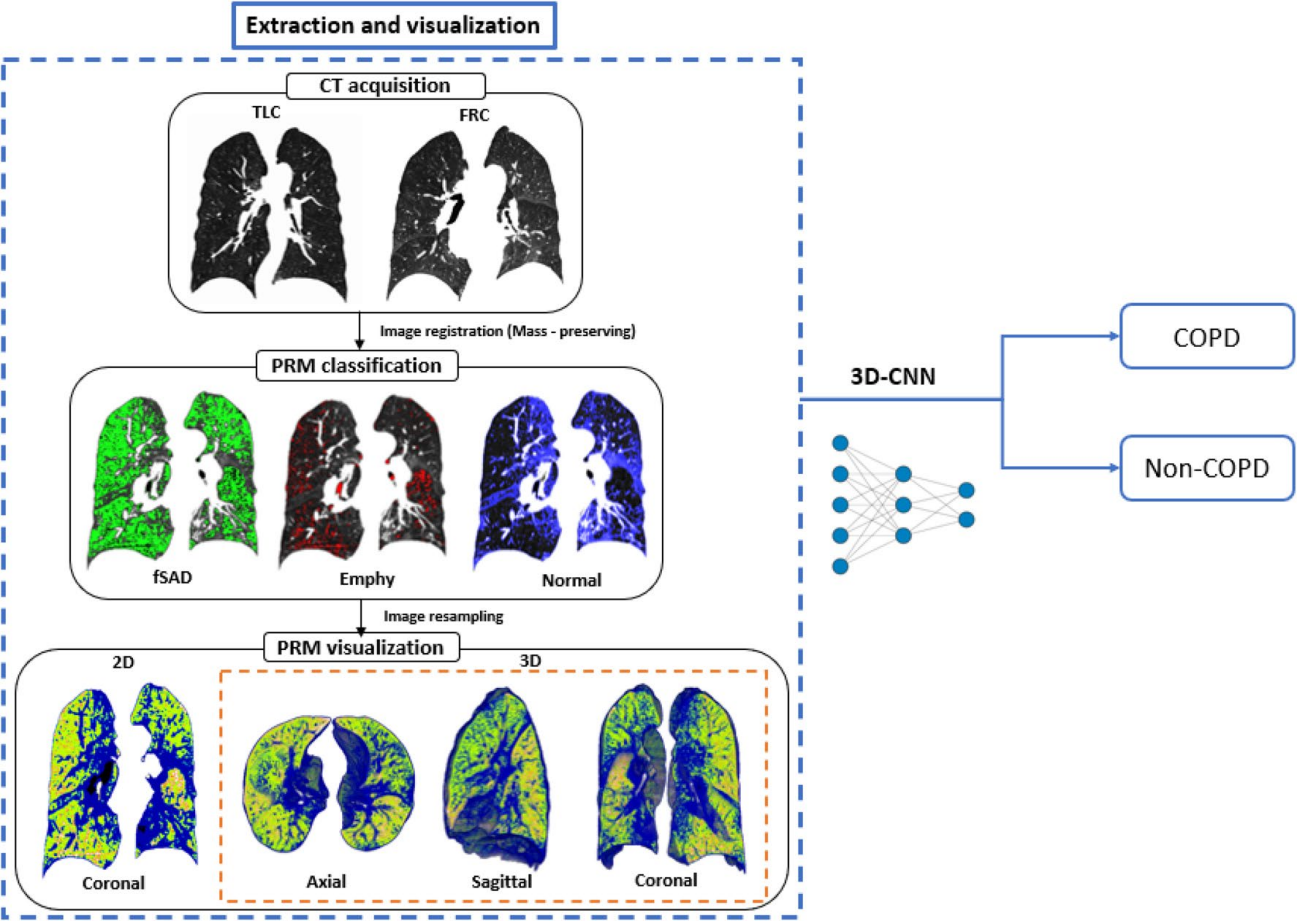
Study design and main experimental produces of 3D-cPRM, operating on a subject with stage III COPD. This figure was generated with ImageJ (version 1.53a, https://imagej.net/) and Microsoft PowerPoint 2010 (version 13328.20292; https://www.microsoft.com).

The lobar variables were computed from the CT images of 596 subjects with a 512 × 512 matrix and image registration, and the imaging metrics were derived from PRM. The lobar variables included the determinant of the Jacobian (*J*, a measure of volume change), the anisotropic deformation index (ADI, the magnitude of directional preference in the volume change), the slab rod index (SRI, the directional preference in the volume change), total lung capacity (TLC), functional residual capacity (FRC), change in air-volume fraction difference (Δ*V*_air_^*f*^, i.e., air-volume difference/voxel size). Imaging metrics obtained from lobar variables, raw 3D CT images [IN (inspiration) and EX (expiration)], and a concatenate of IN and EX were used for classification by CNN. As shown in Fig. 1, our procedure consists of two main steps: CT image processing and COPD/non-COPD classification by 3D-CNN.

### Demographics and lung functions

Demographic, PFTs, and QCT-based lung functions were acquired from COPD and non-COPD subjects (Table 1). The COPD subjects included 84.8% males (average age 72.9 years) with significantly reduced FEV1 and FVC, percentage predicted values, and FEV1/FVC. The percentages of Stage I, II, III and IV subjects on the global initiative for chronic obstructive lung disease (GOLD) scale were 43.1%, 42.7%, 13.2%, and 1.0%, respectively. Meanwhile, the non-COPD subjects were younger females with normal pulmonary functions. The weights were significantly different between the two groups (*p* < 0.001). The mean (SD) radiation dose during the entire CT scanning (scout, IN, and EX imaging) was 9.15 (2.34) mSv per subject. Regarding the QCT-based air volumes, both TLC and FRC were lower in the COPD subjects than in the non-COPD subjects. The percent emphysema lung (Emph%) and functional small-airway disease lung (fSAD%) were significantly larger in the COPD subjects than in the non-COPD subjects (*p* < 0.001). The COPD patients also exhibited a significantly reduced Jacobian, indicating the degree of local deformation^22^, and an increased SRI, indicating the preferential deformation direction^22^.

**Table 1:** Demographics and lung functions derived from the PFTs and QCTs of COPD and non-COPD subjects.

	Non-COPD subjects (*n* = 392)	COPD subjects (*n* = 204)	*P* value
**Demographics**			
Male/females, *n* (%)	190/202 (48.5%/51.5%)	173/31 (84.8%/15.2%)	< 0.001
Age, years	55.9 (16.3)	72.9 (7.4)	< 0.001
Height, cm	161.3 (10.2)	161.3 (7.5)	0.992
Weight, kg	63.2 (12.0)	59.8 (9.8)	< 0.001
Never/former/current smokers, *n* (%)	262/54/76 (66.8%/13.8%/19.4%)	50/53/101 (24.5%/26.0%/49.5%)	< 0.001
COPD stages (I, II, III, IV), *n* (%)	0	88/87/27/2 (43.1%/42.7%/13.2%/1.0%)	
**PFT-based lung functions**			
FEV_1_, %predicted	100.0 (14.1)	75.0(20.0)	< 0.001
FVC, %predicted	98.2 (13.6)	93.1(20.0)	< 0.001
FEV_1_/FVC × 100	80.4 (6.0)	58.9 (8.0)	< 0.001
**QCT-based lung functions**			
TLC, litre	4.7 (1.3)	4.3 (1.0)	< 0.001
FRC, litre	3.0 (1.0)	2.8 (0.9)	0.005
Emph%	1.7 (2.5)	8.5 (7.5)	< 0.001
fSAD%	11.2 (13.4)	23.7 (13.6)	< 0.001
ADI	0.4 (0.1)	0.3 (0.1)	0.074
SRI	0.5 (0.03)	0.6 (0.03)	< 0.001
*J*	1.7 (0.4)	1.5 (0.3)	< 0.001

Values are presented as mean (SD).

QCT, quantitative computed tomography; PFT, pulmonary function test; FVC, forced vital capacity; FEV_1_, forced expiratory volume in 1 s; FRC, functional residual volume; TLC, total lung capacity; Emph%, percent emphysema; fSAD%, percent functional small-airway disease; ADI, anisotropic deformation index; SRI, slab rod index; *J*, Jacobian.

### 3D parametric-response mapping

Figure 1 also shows the spatial PRM distributions in a stage III COPD subject. The total fSAD% was much higher in COPD than in non-COPD subjects (*p* < 0.001) (Table 1). The PRM components were spatially distributed. Quantitative measurements on a PRM spatial model provide physicians with important insights into COPD progression levels (Fig. 1).

### Training and testing results

**Figure 2:**
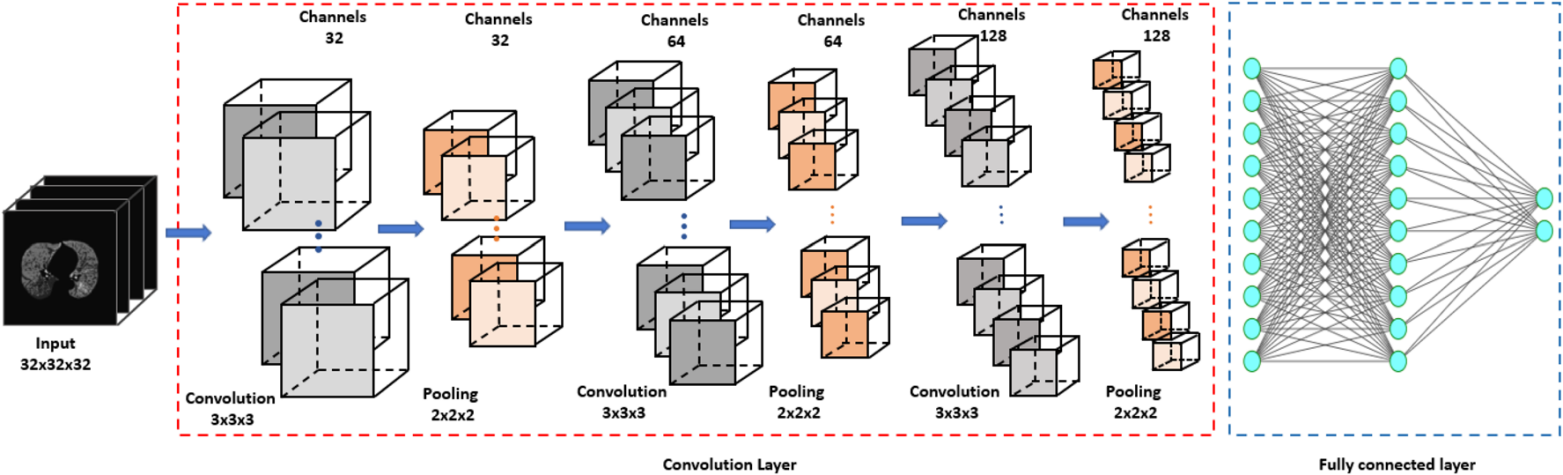
Architecture of the 3D-CNN model. This figure was generated with Microsoft PowerPoint 2010 (version 13328.20292; https://www.microsoft.com).

Figure 2 illustrates the architecture of our nine-layer 3D-cPRM method (the 3D-CNN model is described in the “Methods” section). The accuracies, sensitivities, and specificities of the lung functional variables are presented in Table 2. Both Δ*V*_air_^f^, concatenate of IN and EX, and PRM were better predicted than the other functional variables such as *J*, ADI, and SRI, the raw 3D CT images (IN and EX). The sensitivity was higher for concatenate of IN and EX (95.1%) than for PRM (88.3%) and ΔV_air_^f^ (80.4%), and far beyond that of the original IN image (71.8%).

**Table 2:** Performances of different 3D networks on the PRM dataset, the same 3D-CNN on different input datasets, and different 2D networks on the PRM dataset.

	Accuracy	Precision	Sensitivity	F1	Specificity	AUC score
**Different 3D networks with PRM dataset**						
3D-CNN—Naive model	89.3	82.6	88.3	85.1	93.6	0.937
3D-CNN—DenseNet121	86.9	78.1	85.8	81.2	92.3	0.904
3D-CNN—VGG16	77.4	69.8	58.5	57.4	84.0	0.827
3D-CNN—Resnet50	87.2	79.8	85.8	82.4	92.2	0.906
3D-CNN—InceptionV3	83.9	76.0	72.7	72.8	88.5	0.861
**3D-CNN with different input datasets**						
IN	85.1	80.6	71.8	74.8	88.2	0.900
EX	86.5	75.5	86.9	80.3	93.7	0.907
ΔV_air_^f^	86.6	80.0	80.4	79.9	90.1	0.897
*J*	85.2	76.4	83.3	79.4	91.1	0.895
ADI	83.0	74.1	78.0	75.2	88.8	0.862
SRI	84.9	76.8	80.4	77.7	90.2	0.886
Concatenate of IN and EX CT images	87.2	76.1	95.1	84.0	97.2	0.923
**Different 2D networks with PRM dataset**						
2D-CNN—Naive model	84.8	78.6	71.0	74.6	87.3	0.861
Pretrained—DenseNet121	86.7	77.8	85.4	81.4	92.0	0.899
Pretrained—VGG16	87.5	74.1	97.6	84.2	98.5	0.938
Pretrained—Resnet50	88.3	84.6	80.5	82.5	90.1	0.901
Pretrained—InceptionV3	88.3	80.0	87.8	83.7	93.3	0.923

**Figure 3:**
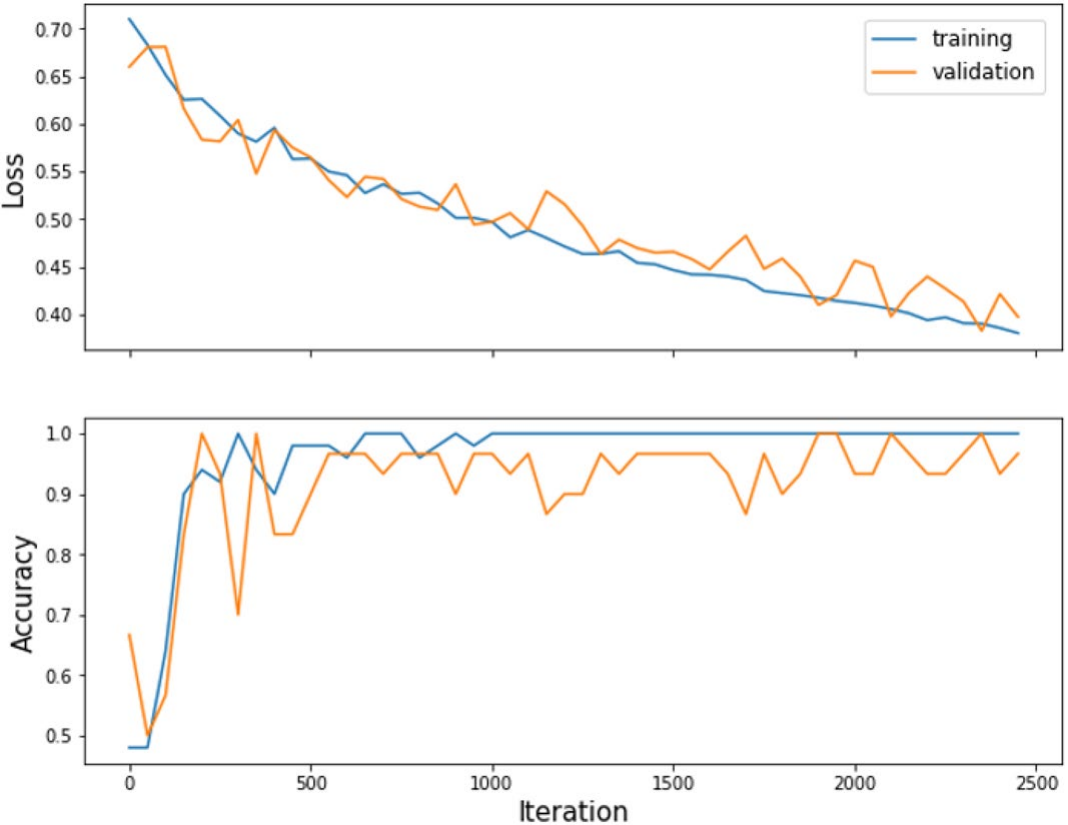
The loss and accuracy of the PRM training and validation datasets.

**Figure 4:**
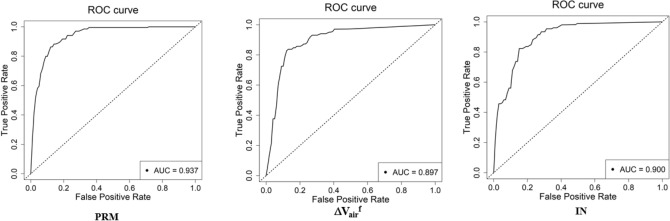
ROC curves of the COPD indices PRM, Δ*V*_air_^f^, and IN.

**Figure 5:**
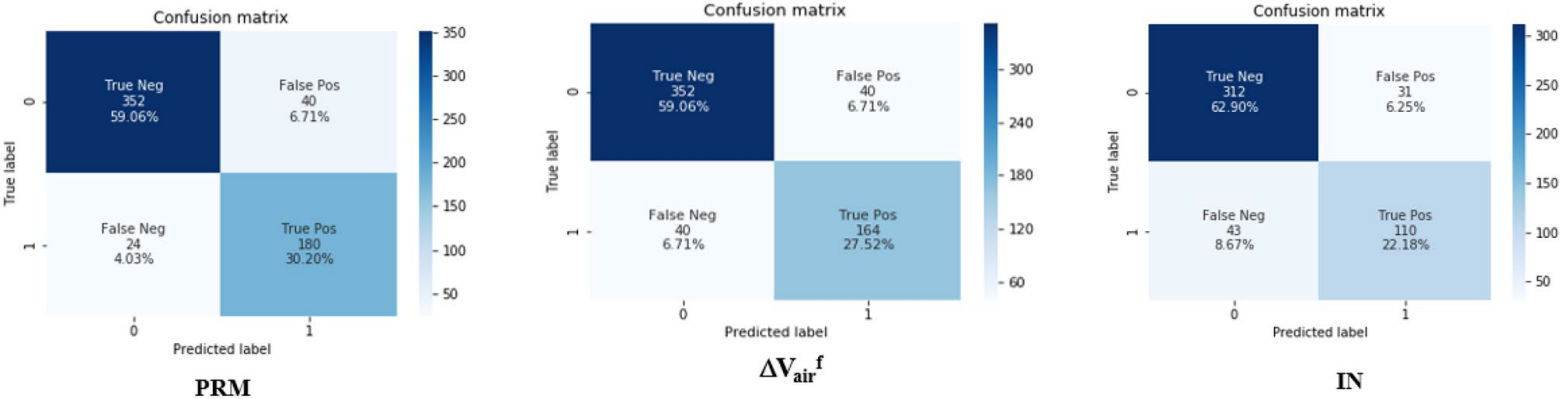
Confusion matrices of PRM, Δ*V*_air_^f^, and IN.

Figure 3 shows the accuracies and losses on the training and validation datasets of 3D-cPRM model as the iterations proceeded. The training loss decreased continuously, reaching approximately zero after 500 iterations; meanwhile, the training accuracy increased gradually to more than 0.95 after 500 iterations. Overall, the accuracy of the validation dataset reached ~ 90% and the corresponding loss was close to zero. When the IN, EX, Δ*V*_air_^f^, *J*, ADI, SRI, concatenate of IN and EX, and PRM were input to the CNN, the prediction accuracy reached 85.1%, 86.5%, 86.6%, 85.2%, 83.0%, 84.9%, 87.2% and 89.3%, respectively (Table 2). The CNN with PRM outperformed the CNNs with the other functional variables, suggesting that 3D images from PRM present more visual differences between COPD and non-COPD lungs than other images. The concatenate of IN and EX and Δ*V*_air_^f^ also achieved relatively high accuracy (87.2% and 86.6%, respectively). Figure 4 shows the receiver operating characteristic (ROC) curves and AUCs of PRM, ΔV_air_^f^, and IN. Consistent with the prediction accuracies, the AUC was highest for PRM (0.937), followed by IN and ΔV_air_^f^ (with AUCs of 0.900 and 0.897, respectively). The confusion matrix of PRM (Fig. 5) reveals 40 false positives (FP, meaning that non-COPD was wrongly predicted as COPD) and 24 false negatives (FN, meaning that COPD was wrongly predicted as non-COPD). Therefore, the sensitivity of the model on the PRM dataset was 88.3%. The FP:FN ratios of Δ*V*_air_^f^ and IN were 40:40 and 31:43, respectively, implying sensitivities of 80.4% and 71.8%, respectively.

### 3D gradient-weighted class activation mapping (3D Grad-CAM)

**Figure 6:**
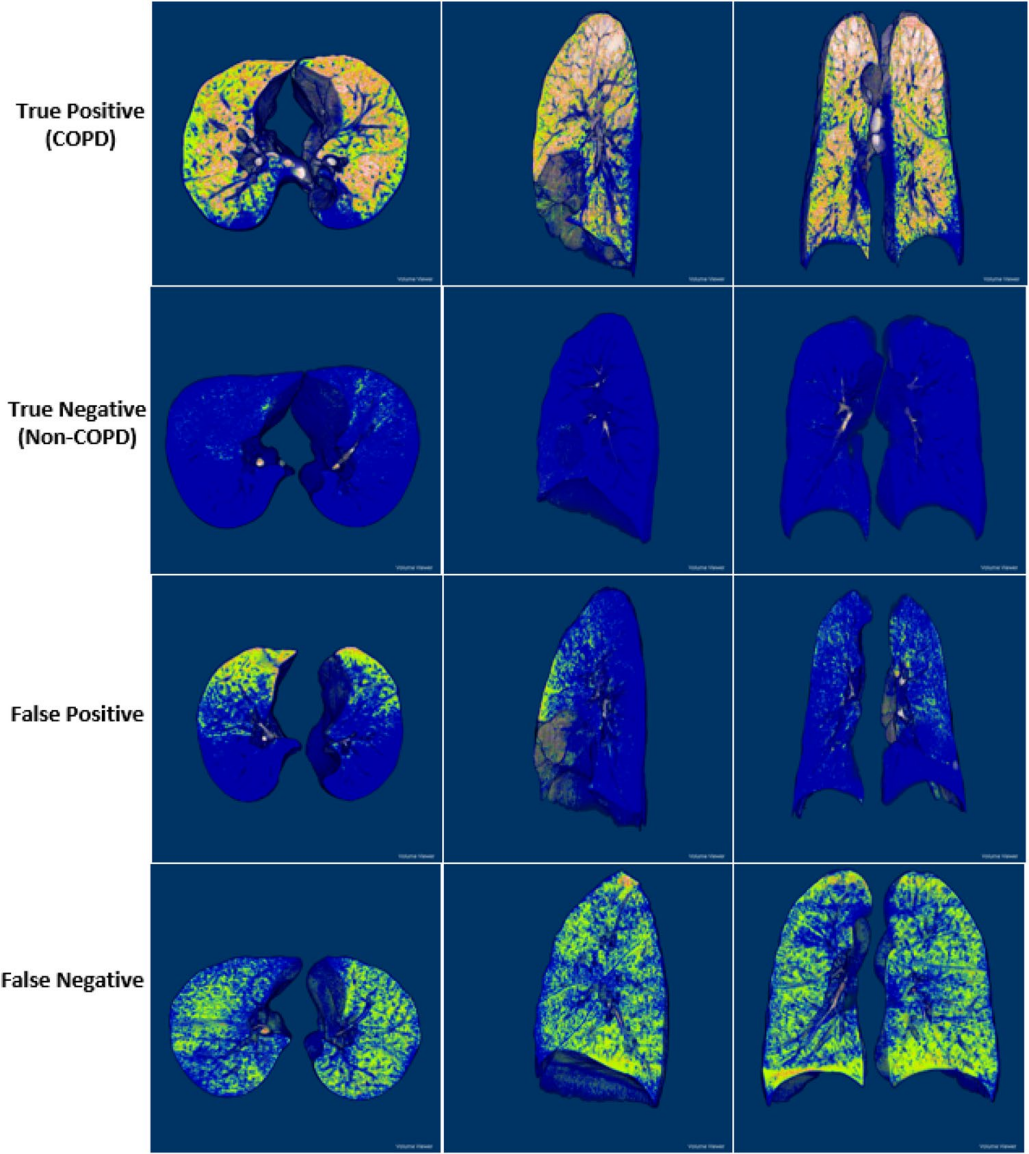
Classification samples of TP (first row), TN (second row), FP (third row), and FN (fourth row) in the axial (left), sagittal (center), and coronal (right) views of 3D PRM images. This figure was generated with ImageJ (version 1.53a, https://imagej.net/) and Microsoft PowerPoint 2010 (version 13328.20292; https://www.microsoft.com).

Figure 6 presents some 3D PRM views of TP, TN, FP, and FN samples in three representative planes (axial, sagittal and coronal) (Fig. 6), obtained by ImageJ open-source software (version 1.53a, National Institute of Health). The four cases show different characteristics of PRM structure: clearly dispersed disease with significant distributions of fSAD and emphysema (TP), unnoticeable disease (TN), clustered disease dominated by fSAD (FP), and dispersed disease with substantial fSAD and negligible Emph (FN). The correctly predicted COPD subjects (first row of Fig. 6) presented more significant fSAD% and Emph% in their lobes than non-COPD subjects (second row of Fig. 6). The appearance differences between FP and FN were very difficult to represent, even by computer vision techniques.

**Figure 7:**
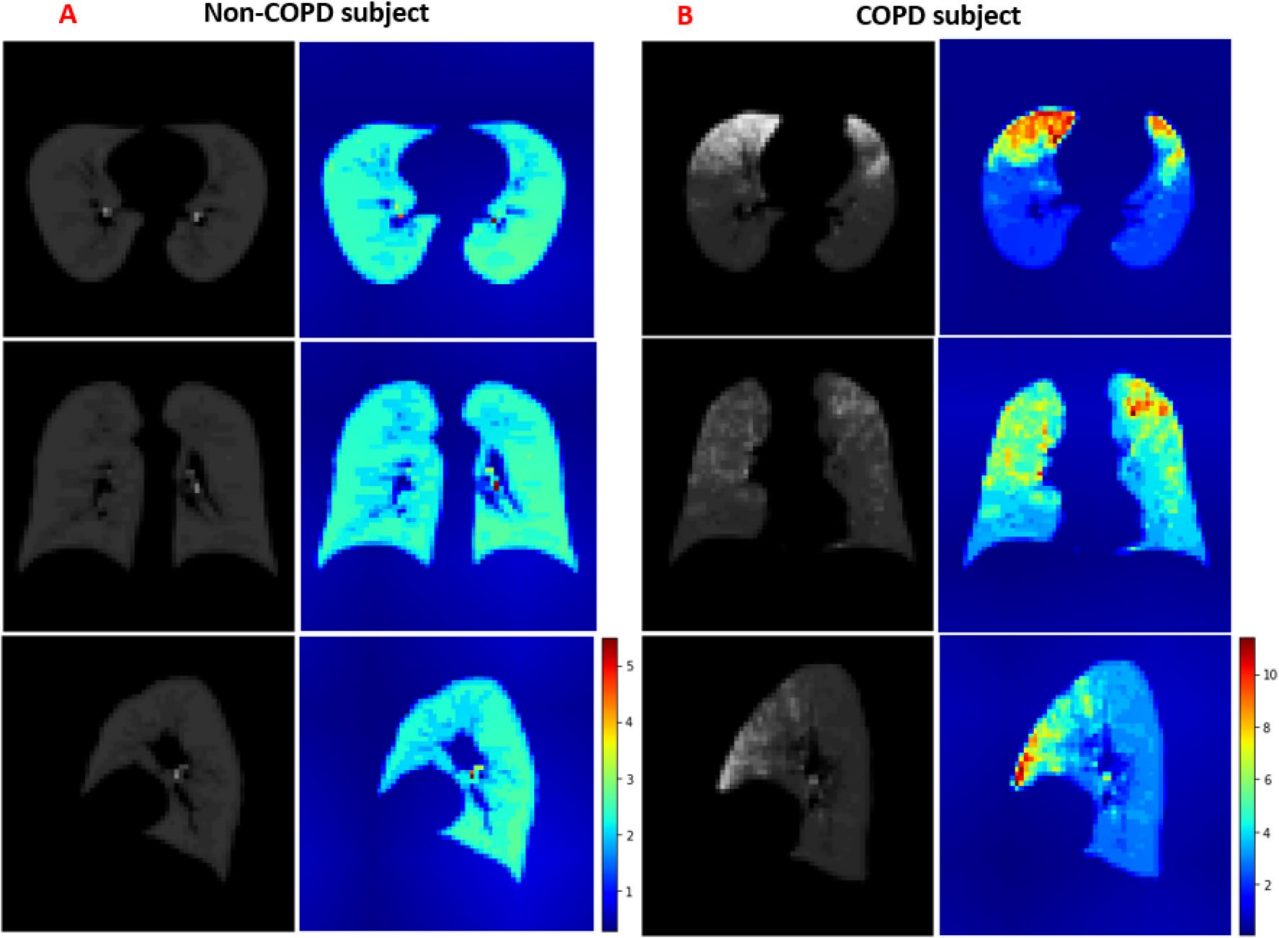
Axial (top), sagittal (center), and coronal (bottom) views of PRM images (gray) and their Grad-CAM heatmap images (color), generated from non-COPD (**A**) and COPD (**B**) subjects. This figure was generated with Microsoft PowerPoint 2010 (version 13328.20292; https://www.microsoft.com).

To overcome the lack of transparency in CNN classification and the drawing of simple conclusions in human diagnosis, we applied Grad-CAM to the trained CNN. The Grad-CAM algorithm visualizes the feature extraction during the learning process of CNN as heatmaps. From the altered images of COPD versus non-COPD, we can extract the useful discriminative features. Different images from the axial, sagittal and coronal views corresponded to different maps in both groups. Abnormal regions in the lung manifested as increased values in the Grad-CAM results, implying that zero values in the heatmap correspond to normal regions in the lung. The heatmaps highlighted the PRM features that discriminated between COPD and non-COPD (Fig. 7). Most of the regions in the non-COPD lung were blue (Fig. 7A), whereas many hot colors appeared in the COPD lung (Fig. 7B). The class-discriminative regions were mainly observed in the upper and middle lobes in the COPD subjects (where the fSAD% and Emph% were also elevated), and at the local parenchyma near the airways in non-COPD subjects. Visualization of the learning process by Grad-CAM revealed that PRM became more focused by CNN as the training progressed.

### Comparing CNN models

The classification performance of our 3D-CNN was compared with those of four successful CNN models: DenseNet121^23^, VGG16^24^, ResNet50^25^ and InceptionV3^26^. For a fair comparison, we converted these 2D models into the 3D domain and trained them on the same input dataset. The DenseNet121, VGG16, ResNet50, and InceptionV3 models consist of many deep convolutional layers, and involve significantly more network parameters than our model. The accuracies of DenseNet121, VGG16, ResNet50, and InceptionV3 in PRM classification were 86.9%, 77.4%, 87.2%, and 83.9%, respectively, confirming that our 3D-CNN model outperforms other typical CNN networks.

Although 2D-CNN with multi-slice representation has been a popular choice in medical image analyses, it unavoidably loses information. Here, we compared the performances of the proposed model to an alternative 2D-CNN model. The accuracy of our 2D-CNN model was 84.8%, versus 86.7%, 87.5%, 88.3%, and 88.3% for pretrained DenseNet121, VGG16, ResNet50, and InceptionV3, respectively. In a natural CNN, the proposed 3D approach significantly outperformed the 2D approach. Therefore, the 3D context is important for differentiating between COPD and non-COPD structures, necessitating the development of 3D pretrained models.

## Discussion

This study developed a deep 3D-CNN model that identifies COPD from CT imaging data of current, former smokers and normal subjects. Among the lung functional variables, the PRM was especially useful for identifying COPD. The PRMs extracted from CT images present distinct and abnormal morphological differences between COPD and non-COPD lungs. A deep CNN better represents these abnormalities from 3D PRM images than from 2D PRM images; in the former case, the classification accuracy of COPD versus non-COPD reached 89.3%. To our knowledge, our method identifies COPD patients at least as accurately as previous classification approaches^17–19,27^. A strong positive correlation was found between some combinations of PRM phenotypes and 3D CNNs.

As a COPD abnormality manifests in various forms and severity, it is not easily representable in the complex 3D structure of a human lung. COPD-induced abnormalities in the lung functional variables are multidimensional^28^ and vary with anatomical location. Furthermore, quantitative measurements of the lung functional variables of COPD are non-standardized. The uncertainties and difficulties are enhanced by the different acquisition and reconstruction protocols of different CT scanners^29–31^. Our 3D-cPRM method avoids these difficulties by efficiently distinguishing PRM abnormalities in COPD. The developed CNN fully exploits the available CT data, rather than pre-selecting 2D slices or snapshot images for 2D CNNs^18^, or directly inputting CT slices to a 2D-CNN^17^.

Table 3 overviews the datasets, methods, and classification results of some recent deep-learning studies that distinguish COPD from non-COPD subjects. The use of different datasets and methods precludes a fair comparison, but all models yielded satisfactory results. The advantage of the proposed 3D-cPRM approach, which achieved a detection accuracy of 89.3%, was confirmed above. Our result is comparable to that of a previous CNN study^17^ and far exceeded that of multi-view snapshots of a 3D lung-airway tree (24/596 FPs in our method, vs. 26/280 FPs and an accuracy of 88.6%^18^). Therefore, a 3D-CNN can effectively eliminate the false-positive results generated by 2D-CNN. The 3D-cPRM method (with a sensitivity of 88.3%; see Fig. 5) reduced the number of FPs from 43 in the 2D-CNN to 24. The accuracy of the pretrained models approached that of the 3D-CNN model, and surpassed that of the natural 2D-CNN model. This implies that a pretrained 3D model can improves the prediction accuracy of future COPD cases. This significant improvement again demonstrates that 3D-cPRM can clinically distinguish between the PRM characteristics of COPD and non-COPD subjects. When inputted with the combined 3D IN and EX images, our model achieved 95.1% sensitivity and 97.2% specificity, exceeding that of the PRM input (88.3% sensitivity and 93.6% specificity; see Table 2). However, this input combination slightly reduced the accuracy from that of PRM (87.2% vs. 89.3%), and doubled the computational cost from that of PRM (42 min vs. 80 min).

**Table 3:** Previously published classification results of COPD versus non-COPD datasets.

Related work	Data	Methods	Accuracy (%)
González et al.^17^	Original CT slices of COPDGene testing cohort (*n* = 1000)	2D-CNN	77.3
Ran Du et al.^18^	Multi-view snapshots of 3D lung-airway tree (190 COPD—90 Non-COPD)	2D-CNN	88.6
Ran Du et al.^18^	3D airway trees (190 COPD—90 Non-COPD)	3D-CNN	78.6
Feragen et al.^19^	Airway trees (980 COPD and 986 Non-COPD subjects)	SVM	64.9
Xu et al.^27^	1/6 of the total height (z) of the original CT sequences (190 COPD and 90 healthy control subjects)	Deep CNN transferred multiple instance learning (DCT-MIL)	99.3
Our work	3D PRM	3D-CNN	89.3

Our model was supported by the Grad-CAM implementation, which distinguished between the COPD and non-COPD imaging features in the PRM input. The heatmap images of Grad-CAM were of limited usefulness when the highlighted class-discriminative regions were unclear. This can be explained by the low resolution of our convolutional layer (32 × 32 × 32 voxels). Smaller kernels in Grad-CAM yield poor contrast and visualization. Most of the regions in non-COPD lungs were blue, whereas hot-color regions were observed in the COPD subjects. This result reveals that (unlike humans) deep CNNs can memorize large volumes of CT image information. Regardless of the classification method, the CNN identified the PRM as an important imaging feature of COPD. The PRM was more significantly correlated with COPD than the other functional variables (Δ*V*_air_^f^, SRI, ADI, and *J*), affirming that this variable both discriminated and predicted the clinical features of COPD. The trained CNN automatically and objectively identified the PRM as an important feature during the COPD versus non-COPD discrimination. By visualizing the classification target of the CNN, Grad-CAM overcame the common drawback of deep-learning models, namely, the non-transparent interpretation. Unlike current image processing methods that require the summary statistics on a feature of interest, deep learning uses all data available in the image, and predicts the clinical relevance at the large-population level.

Our study has several limitations. First, our datasets were sourced from two hospitals adopting similar imaging protocols and the same image analysis. However, various restructured kernels are mainly responsible for changes in Hounsfield (HU) values. These variations affect the PRM classification maps and the subsequent calculation results. The scanners in our study used the same B3_f kernel, differing only in the 0th and 5th generations. If the reconstruction kernels and scanners are inconsistent (a common complication of multi-center data), the input parameters must be normalized into a similar data range for accurate classification. Second, the image noise and registration errors may also affect the accuracy of PRM map prediction. Errors in the registration technique are a common problem for all CT-based quantitative methods^32^. Here we employed a mass-preserving registration method that maintains the same tissue volume between two inflation levels. This approach has been rigorously validated in lungs undergoing large deformations^33^. An image registration algorithm using this approach achieved sub-voxel accuracy^33^. After downsampling our imaging data, the resampled voxel size was much larger than the spatial accuracy, reducing the likely impact of the image registration accuracy on the prediction accuracy of our deep-learning model. Finally, in a clinical setting, the classification of using CT-based features may not be used directly, because we did not associate any implication of CT-based features with clinical measures. This limitation should be supplemented with a well-designed prospective study that collect clinical and therapeutic information, as well as CT images.

In this study, we demonstrated that 3D PRM is a versatile imaging biomarker of phenotypic COPD. In particular, 3D PRM localizes the COPD disease states (fSAD and emphysema) in high volume. Unlike other methodologies, 3D PRM easily provides the detailed spatial information on the distribution and location of COPD disease. By studying the unique structural and functional features between the two population groups, physicians can tailor their therapeutic interventions to individual patients with COPD, complementing standard clinical techniques. Especially when a PFT diagnosis is uncertain, COPD is commonly detected by QCT. Our diagnostic 3D-cPRM method is a useful initial tool for investigating COPD in a limited number of patients. However, a good deep diagnostic model should be able to distinguish COPD from other lung diseases (such as asthma and lung cancer), and assess the severity of COPD (GOLD stages I–IV). In the next phase of our study, we will enroll more patients from different centers and update the dataset to achieve stable model performance. By adapting more advanced methodological strategies based on CNN models, we could precisely distinguish COPD from non-COPD in CT images^34^. Furthermore, the high extraction ability of 3D PRM imaging ensures good contrast. In recent CNN studies, the accuracy has been enhanced by combining different imaging modalities^35,36^. Although the 3D-CNN inputted with PRM CT images performed comparably to previous findings, its accuracy might be enhanced by combining ultrasound, X-ray, magnetic resonance imaging, single photon emission computed tomography, or positron emission tomography. The effectiveness of these modality combinations should also be considered in future work.

In conclusion, alongside the worldwide prevalence and impact of COPD, quantitative image visualization has become a cornerstone of clinical investigation. We proposed a deep 3D CNN that automatically identifies COPD from the lung functional variables, and visualizes the results. COPD manifests as abnormal appearances of the lung parenchyma, which were well represented by the deep CNN. The proposed method will enable early-stage identification of COPD, thereby reducing the missed-diagnosis rate. It can also elucidate the underlying disease pathogenesis and improve the management of COPD, especially in a time-constrained clinical environment. Our results clarified the potential of the PRM extraction features in COPD classification. The analysis of deep training processes, combined with specific imaging characteristics, can facilitate the discovery of new medical features.

## Methods

### Dataset

All methods complied with the guidelines and regulations of the participating centers. Informed consent was obtained from all participating subjects. All procedures were approved by the Institutional Review Board of Kangwon National University Hospital (KNUH) and Jeonbuk National University Hospital (JNUH) at the individual sites (KNUH 2019-06-007 and CUH 2016-03-020-005). The 596 subjects (204 COPD, 392 non-COPD) were enlisted from subjects who underwent CT scans in KNU and JNU Hospitals. The KNUH subjects had a clinically tested disease history of asthma, pneumonia, and abnormal pulmonary function. The acquisition and reconstruction parameters of the two CT scanners are given in Table 4. The KNUH subjects included 50/53/101 COPD subjects with never/former/current-smoker status accompanied by cement dust effects. Their data were supported by a Korean research project called the Chronic Obstructive pulmonary disease in Dusty Areas near cement plants (CODA), which monitored this cohort over 10 years. The JNUH subjects (control group) included 262/54/76 non-COPD subjects with never/former/current-smoker history, and no or little exposure to cement dust. Their data were collected by JNUH during 3 years^37^. Both CT scanners had similar specifications and imaging protocols (see Table 1) and used the same filtered back-projection reconstruction kernel (Siemens Definition Flash 128 slices B30f. and Siemens Definition AS 64 slices B35f.).

**Table 4:** Scanners and scanning protocols used on the COPD and non-COPD subjects.

Institution (subjects, *n*)	JNUH (296/596)	KNUH (300/596)
Scanner manufactory	Siemens definition flash 128 slices	Siemens definition AS 64 slices
Scan type	Spiral	Spiral
Rotation time(s)	0.5	0.5
Detector configuration	128 × 0.6 mm	64 × 0.6 mm
Pitch	1	1
Peak kilo voltage, kVp	120	140
Exposure (mAs)	110, Effective for inspiration 50, Effective for expiration	100, Effective for both inspiration and expiration
Dose modulation	Care dose OFF	Care dose OFF
Reconstruction algorithm	B35f.	B30f.
Thickness (mm)	1	0.6

JNUH, Jeonbuk National University Hospital; KNUH, Kangwon National University Hospital; mAs, milliampere-seconds.

### Registration

By registering the images of two or more lung CT images obtained at different static lung volumes (typically, the inspiration volume and one or more expiratory lung volumes), we obtain the functional variables of the images, such as the regional ventilation variable (distribution of the inspired air bolus). The pulmonary registration technique calculates the optimal local-to-local correspondence between images captured with different modalities or collected at different times^38^. The similarity measure of the sum of squared tissue volume difference (SSTVD)^38^ has been demonstrated to successfully improve registration of lung images with large deformation. This approach computes the changes in the reconstructed HUs caused by the inflationary lung motions. For reference, the attenuation values of air, water, and tissue are − 1000, 0 HU, and 55 HU respectively. Prior to registration, lung parenchyma, airway, and vessel analyses were automated using VIDA Pulmonary Workstation and Apollo software (VIDA Diagnostics, Coralville, IA) for extracting their binary-mask segmentation images. The binarized images were registered using a segmentation and registration toolkit ITK (https://itk.org/) implemented in a homemade C++ program. In this paper, the paired CT data were registered with SSTVD^22,38–40^. The parenchymal/global functional variables (Δ*V*_air_^f^, *J*, ADI, and SRI), were extracted by an imaging registration technique applied between the inspiration and expiration images. The Emph%, fSAD%, and Δ*V*_air_^f^ of the lungs were assessed by a CT density-threshold method, whereas the ADI, *J*, and SRI were assessed by the mechanical strains estimated in the image registration (Table 4). The PRM images were trained to compare the raw inspiration and expiration CT images, and the Δ*V*_air_^f^, ADI, *J,* and SRI values were generated from the lung function parameters.

### Parametric response mapping

The COPD phenotypes were classified by their topological properties obtained by PRM analysis of the inspiratory (reference)/expiratory (floating) lung CT images. The PRM created from the paired CT lung images improves the COPD phenotype detection by allowing visualization and quantification of the fSAD and Emph components^4^. The registered image voxels (discrete 3D image units consisting of the inspiratory and expiratory attenuations in HUs) within the segmented lung volume were classified into three categories by imposing two thresholds: − 950 HU on the inspiratory CT and − 856 HU on the expiratory CT. The PRM components were colored as follows: 32 (fSAD, color-coded green; >  − 950 HU on IN and ≤  − 856 HU on EX) for functional small-airway disease; 64 (Emph, color-coded red; ≤  − 950 HU on IN and ≤  − 856 HU on EX) for emphysema clusters, and 8 (normal, color-coded blue; > − 950 HU on IN and >  − 856 HU on EX) for healthy lung parenchyma and unclassified voxels.

### 3D convolutional network model

When selecting a 3D-CNN for medical imaging classification problems, one must consider the high computation time, large size of the training datasets, and lack of pretrained models. These difficulties have been ameliorated by different CNN architectures. Unlike 2D CNNs, a 3D-CNN can encode representations of volumetric fields, and therefore extract more discriminative features via the 3D spatial information. Our 3D-cPRM mainly consists of 3D convolutional layers, 3D pooling layers, and fully connected layers, which are successively stacked into a hierarchical architecture. Each channel in a 3D-CNN is a 3D feature volume rather than a 2D feature map. The convolutions and max-pooling layers of a 3D-CNN are operated in a cubic manner. In a 3D max-pooling operation, the maximum value in the cubic neighborhood is selected and input to the subsequent feature map. After the pooling operation, the resolution of the feature volumes is reduced by an amount corresponding to the pooling kernel size. Theoretically, the pooled learned features are invariant to local translations in 3D space, which is eminently useful for image processing^41^.

### CNN architecture

Considering the three-dimensional processing, size of our training data, and available GPU computational power, we constructed a simple architecture for computational efficiency without overly compromising the accuracy. The network was built within the deep-learning framework Keras, and the input images were read by SimpleITK, an open-source multi-dimensional image analysis program. Our 3D deep network (see Fig. 2) consisted of 9 layers: 3 convolutional layers, 3 batch normalization layers, and 3 max-pooling layers. The datasets were resampled to 32 × 32 × 32 voxels using the linear interpolation method, and the HU of each pixel was normalized within the range [0, 1]. After each convolutional layer of kernel size 3 × 3 × 3, the feature volumes were down-sampled by a max-pooling layer with a 2 × 2 × 2 voxels window. Finally, three fully connected layers (two with 128 neurons and one with 2 neurons) preceded the classification layer. As the activation function, we employed a rectified linear unit (ReLU) in each fully connected and convolutional layer. The model was trained from scratch with weights initialized from a normal distribution with mean *μ* = 0 and standard deviation *σ* = 0.1. The number of filters was determined as 32, 64, and 128 according to experiences. The COPD and non-COPD labels were then distinguished by a softmax function. The neural network was trained with the binary cross-entropy between the predicted and true diagnoses as the loss function. The convolution network structure was optimized by an Adam optimizer with the default learning rate (0.0001)^14,42^. Our CNNs were trained over 2500 iterations on a batch size of 50 samples. The iterative accuracies and losses on the training and testing datasets were plotted to validate the iteration number.

For comparison with other 3D-CNN models, we also implemented a 2D-CNN model by extracting one slice per subject at the 50% location of all slices. The 2D images were augmented with randomly rotated, scaled, sheared, and translated images within a defined range. The original size of the 2D CT image (512 × 512 pixels) was down-sampled to the input size of the 2D-CNN model (256 × 256 pixels). The number of filters was imposed as 32, 64, and 128, consistent with the 3D model, and the original HU value was normalized to between zero and one. We also assessed the 2D pretrained CNN models on the imaging data. For predicting COPD cases among our imaging data, we replaced the fully connected layer of the pretrained DenseNet121, VGG16, ResNet50, and InceptionV3 CNNs with a new fully connected layer, which was trained only on our 2D imaging data.

### 3D gradient-weighted class activation mapping (3D Grad-CAM)

The classification conclusions of CNN models are non-transparent and cannot provide intuitive reasoning and explanations like human diagnostic experts^42^. Here, we adopted a Grad-CAM approach for visualizing the CNN learning process. This method creates a 2D spatial heatmap of the image indicating the focal points of the CNN predictions. The heatmap tracks the spatial attention of the 3D-CNN when predicting COPD disease.

### Training and performance evaluation methods

The performances of the CNNs were evaluated by five-fold cross-validation. One-fold was reserved as the test dataset and the other four folds were used as the training dataset. The CNNs were implemented on an Intel, Xeon, CPU E5-2640 v4 @ 2.40 GHz (20 CPUs) with an NVIDIA GeForce RTX 2080Ti. The classification performance was assessed by the accuracy, precision, sensitivity, F1 score, specificity, confusion matrix, ROC curve, and AUC. These metrics were computed from the true positive (TP), true negative (TN), false negative (FN), and false positive (FP) results^24^. Statistical comparison of the demographics and lung functions was performed by two-sample t-test.
